# Supplementary material for: Identification of Trypanosoma cruzi Growth Inhibitors with Activity In Vivo within a Collection of Licensed Drugs
Source: Microorganisms. 2021 Feb 16;9(2):406. doi: 10.3390/microorganisms9020406 (PMC7920067; doi:10.3390/microorganisms9020406)
Supplement: Supplementary file 1 [file microorganisms-09-00406-s001.pdf]

Supplementary Materials

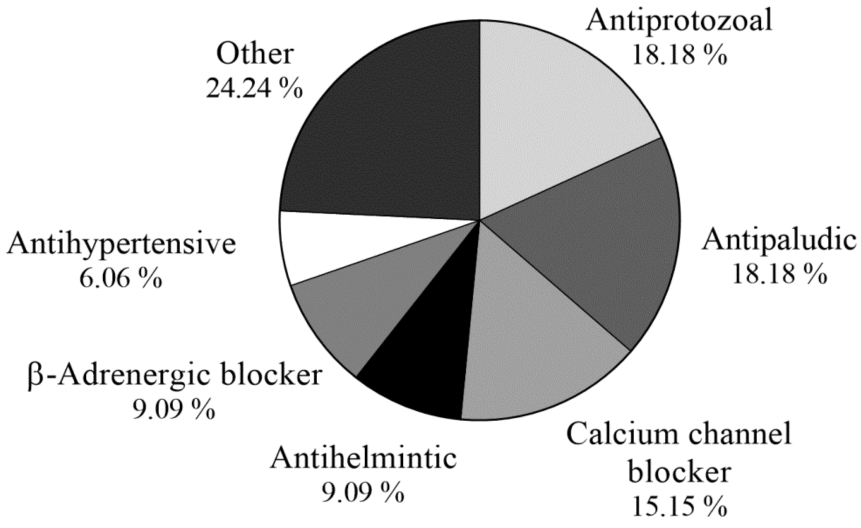

Figure S1. Classification of drugs per class.

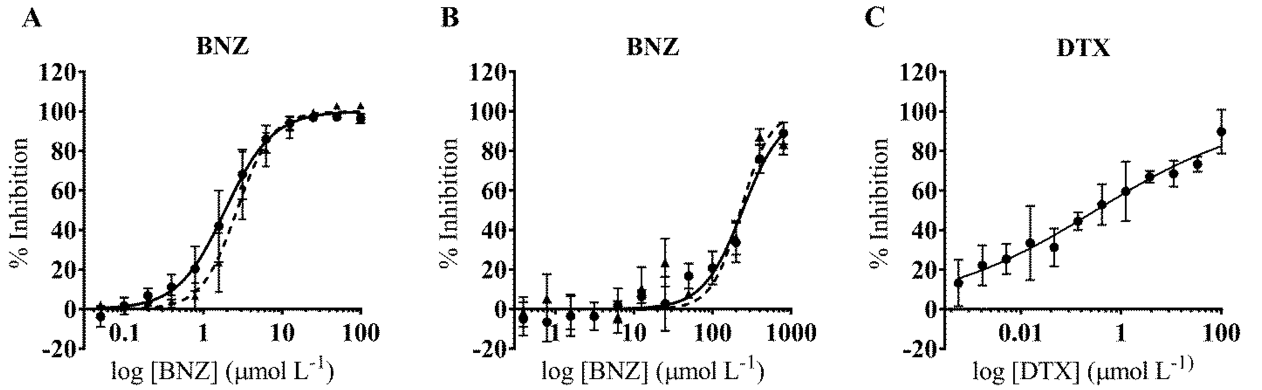

Figure S2. BNZ and DTX dose-response curves. (**A**) BNZ anti-*T. cruzi* activity in the primary assay (circles and straight line) and in the anti-amastigote assay (triangles and dashed line); (**B**) BNZ toxicity in Vero cells (circles and straight line) and HepG2 cells (triangles and dashed line); (**C**) DTX toxicity in HepG2 cells.

Table S1. Average IC_{50} values of non-progressed drugs.

Trade name	Active ingredient	IC_{50} (µmol L^{-1})
Amlodipine Normon	Amlodipine	63.47
Apocard	flecainamide	N.A.
Atenolol Normon	atenolol	>1,000
Biocoryl	procainamide	N.A.
Bisoprolol Normon	bisoprolol	N.A.
Daraprim	pyrimethamine	16.07
Defitelio	defibrotide	N.A.
Diamicron	gliclazide	N.A.
Enalapril Normon	enalapril	N.A.
Eskazole	albendazole	10.46
Glucantime	meglumine antimoniate	15.36
Glucophage	metformin hydrocloride	N.A.
Hemovas	pentoxifylline	194.8
Humatin	paramomycin	N.A.
Iver P (ELEA)	ivermectin	48.33
Quinine sulfate (Hospital Clinic)	quinine sulfate	15.40
Lomper	mebendazole	7.19
Masdil	diltiazem	>1,000
Menaderm	beclomethasone dipropionate - clioquinol	5.81
Nerdipina	nicardipine	65.34
Primaquine (Hospital Clinic)	primaquine	N.A.
Riamet	artemether - lumefrantine	8.23
Solgol	nadolol	374.7
Sotapor	sotalol	N.A.
Tricolam	tinidazole	203.9

N.A., not adjusted; * and # indicate IC_{50} values expressed as drug % per well (v/v) and µg per well, respectively.
